# Associations Between Environmental Conditions and Infection With Respiratory Syncytial Virus in Japan: A Spatiotemporal Analysis

**DOI:** 10.1093/ofid/ofaf392

**Published:** 2025-08-13

**Authors:** Jingyi Liang, Saturnino Luz, You Li, Harish Nair

**Affiliations:** Centre for Global Health, Usher Institute, Edinburgh Medical School, University of Edinburgh, Edinburgh, UK; Centre for Medical Informatics, Usher Institute, Edinburgh Medical School, University of Edinburgh, Edinburgh, UK; Centre for Global Health, Usher Institute, Edinburgh Medical School, University of Edinburgh, Edinburgh, UK; National Vaccine Innovation Platform, School of Public Health, Nanjing Medical University, Nanjing, China; Centre for Global Health, Usher Institute, Edinburgh Medical School, University of Edinburgh, Edinburgh, UK; National Vaccine Innovation Platform, School of Public Health, Nanjing Medical University, Nanjing, China; MRC/Wits Rural Public Health and Health Transitions Research Unit (Agincourt), School of Public Health, University of the Witwatersrand, Johannesburg, South Africa

**Keywords:** environmental health impact, interpretable machine learning, respiratory syncytial virus, spatiotemporal modeling

## Abstract

**Background:**

Respiratory syncytial virus (RSV) poses a significant disease burden among children <5 worldwide. Yet systematic analyses of how complex environmental factors are associated with RSV transmission are still lacking in many countries.

**Methods:**

We introduced a novel 3-stage, data-driven framework to assess the impacts of environmental factors, including meteorological conditions, air pollutants, and extreme weather, on RSV infections from a spatiotemporal perspective. It includes (1) spatiotemporal patterns of RSV transmission; (2) a hierarchical model (HSDLNM) to examine associations between environmental factors and RSV transmission, estimating relative risks (RRs) and 95% confidence intervals; and (3) an interpretable machine learning model, Gaussian Process Boosting, to predict RSV infections using historical environmental data. We validated the applicability of the proposed framework in Japan.

**Results:**

Weekly data on the number of newly lab-confirmed RSV-positive cases, meteorological factors, and air pollutants were collected from 47 Japanese prefectures (2013–2019). We identified the meteorological thresholds strongly linked to elevated RSV infections, particularly weekly average temperature <10°C (RR, 1.10) or >20°C (RR, 1.13) and weekly average relative humidity <60% (RR, 1.04) or >70% (RR, 1.06). Short-term exposure to particulate matter of 2.5 $\mu m\;(PM_{2.5})$ is associated with elevated infection risk. Additionally, historical environmental data aid in forecasting RSV activities in Japan.

**Conclusions:**

This study presents the relationships between environmental factors and RSV infections in Japan. Our framework could be applied to areas with similar RSV seasonality to further understand environmental impacts regionally. This research helps inform policy decisions on RSV prophylaxis strategies, supporting cost-effective measures for controlling and preventing early transmission.

Respiratory syncytial virus (RSV) represents a significant public health challenge worldwide, primarily affecting children <5 years and constituting the leading cause of acute lower respiratory tract infections in infants [1–3]. Understanding the seasonality, geographic distribution, and potential drivers of RSV infections, such as environmental factors, population density, living conditions, etc., is pivotal to understanding transmission and making plans for timely prevention [4]. Unraveling the complex relationships between environmental conditions, including meteorological factors and air pollutants, and RSV transmission is essential for comprehending the seasonality of RSV epidemics. The role of meteorological factors in affecting RSV activity worldwide has been explored [5–8]. Additionally, short-term exposure to air pollutants, such as ozone, particulate matter, and indoor air pollutants, has been linked to respiratory viral infections, including RSV [9–11].

In Japan, ∼25% of RSV patients <2 years old, particularly infants <6 months, require hospitalization due to severe RSV infections [12]. This underscores the urgent need for effective prevention strategies. A comprehensive understanding of RSV seasonality in Japan could help formulate RSV prevention strategies [13]. Recent evidence indicates a shift in RSV epidemic seasonality in Japan since 2017, from autumn and winter to summer and autumn [14, 15]. Additionally, evidence suggests that RSV epidemics could be influenced by environmental factors. A recent study established the nationwide relationship between RSV activity and its meteorological drivers in Japan, affirming that high ambient temperatures and relative humidity are associated with increased RSV infections [16]. At the prefecture level, Onozuka et al. [17] highlighted the positive impact of the diurnal temperature range on RSV cases in Fukuoka.

Collectively, current research illustrates the significant role of environmental factors in influencing RSV activity in Japan. However, key research gaps remain. First, the effect of complex environmental conditions, including both meteorological factors and air pollutants, on RSV transmission is poorly understood. Current studies have mainly focused on analyzing the impact of meteorological factors on RSV transmission. Second, widely applied quantitative methods to analyze such relationships often ignore the spatial heterogeneity and temporal dependency that exist in disease data, potentially leading to biased or spurious relationships [18].

This study aimed to investigate how environmental conditions, including meteorological factors and air pollutants, are associated with the spatiotemporal transmission of RSV in Japan by using an innovative 3-stage framework designed to analyze spatiotemporal data more effectively. We examined the associations between meteorological factors, air pollutants, and RSV cases, affirmed by sensitivity analyses to validate the reliability and robustness of our findings.

## METHODS

### Data

#### RSV Cases

Publicly available RSV data, the weekly number of lab-confirmed RSV-positive cases from the first week of 2013 to the 52nd week of 2019, were extracted from the Infectious Diseases Weekly Report of Japan, Japan's National Institute of Infectious Diseases (NIID) [19].

#### Environmental Data

Meteorological and air pollutant data were extracted from 2 primary sources for the period between January 1, 2013, and December 31, 2019. Meteorological data, including daily metrics of maximum temperature (°C), minimum temperature (°C), average temperature (°C), total precipitation (mm), average relative humidity (%), average visibility (km), average wind speed (km/h), and average sea level pressure (hPa), were extracted from the National Oceanic and Atmospheric Administration (NOAA) [20]. Air pollutant data, including daily mean concentrations ($\mu g/m^{3}$) of nitrogen dioxide ($NO_{2}$), sulfur dioxide ($SO_{2}$), carbon monoxide (CO), and fine particulate matter with a diameter <2.5 μm ($PM_{2.5})$, were extracted from the Japan National Institute for Environmental Studies' atmospheric environment database [21].

Daily environmental data were aggregated at the weekly level. We aggregated weekly precipitation by summation, whereas other parameters were aggregated by averaging. EXtreme Gradient Boosting (XGBoost) models were used to impute the missing values of environmental factors. The data set was randomly split into training (80%) and testing (20%) subsets. The XGBoost models were fine-tuned using grid search and 5-fold cross-validation on the training data, achieving high performance with R-squared ($R^{2}$) values of 0.97 for weekly maximum temperature, 0.98 for weekly minimum temperature, and 0.85 for weekly mean CO concentration on the testing data set (Supplementary Data, page 3).

To explore the impact of weekly temperature fluctuations on RSV transmission, we calculated the weekly temperature range using the weekly maximum temperature and minimum temperature. Additionally, to investigate the association of extreme temperatures and RSV infections, we calculated the weekly number of days with daily average temperatures exceeding 24°C, 30°C, and 35°C, as well as those below 6°C, 0°C, and −5°C.

### Study Design

**Figure 1.**
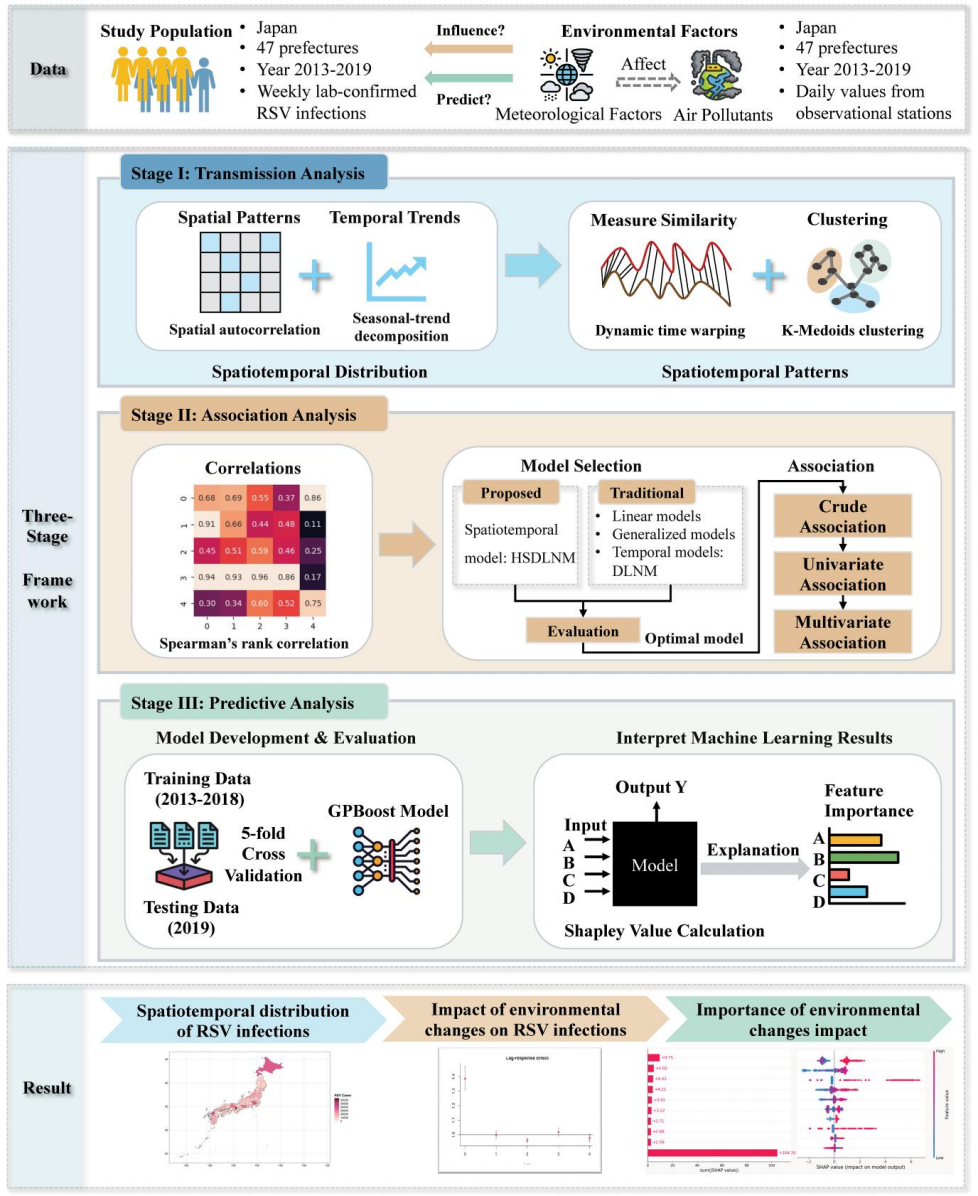
Flowchart of the proposed 3-stage spatiotemporal analytical framework. Abbreviations: DLNM, distributed lag nonlinear model; HSDLNM, hierarchical spatially distributed lag nonlinear model; RSV, respiratory syncytial virus.

We proposed a 3-stage framework to assess the nationwide associations between environmental conditions and RSV cases at the prefecture level in Japan from 2013 to 2019 (Figure 1).

The first phase analyzes the spatiotemporal patterns of RSV cases. Spatial autocorrelation, assessed via Global Moran's *I* [22], quantifies RSV spatial patterns. Seasonal trend decomposition using locally estimated scatter smoothing (LOESS; STL) [23] reveals temporal patterns, including seasonal and long-term trends in RSV infections. Dynamic Time Warping (DTW) [24] was applied to measure the similarity between RSV case trajectories across the 47 prefectures, accounting for potential time shifts in the time-series data. We used K-Medoids [25] to cluster similar RSV trajectories due to its effectiveness in handling noise and outliers. The optimal number of clusters was determined using the elbow method [26].

The second phase assesses the association between environmental variables and RSV infections. We utilized Spearman's rank correlation coefficient [27] to evaluate nationwide correlations between RSV cases and environmental variables. Subsequently, we proposed the hierarchical spatially distributed lag nonlinear model (HSDLNM) to account for the inherent traits of spatiotemporal data. Given that the average incubation period of RSV ranges from 2 to 8 days [28], we applied this model to investigate the lag effect up to 3 weeks on the impact of meteorological factors and air pollutants on RSV cases, respectively, across 47 prefectures in Japan. Before performing regression analysis, we excluded values above the $99^{\mathrm{th}}$ percentile for each parameter to minimize the impact of outliers in the air pollutant data while preserving most of the original data (Supplementary Table 1). Additionally, we selected appropriate basis functions to represent the nonlinear exposure–response relationships, ensuring their accuracy and interpretability (Supplementary Data, page 3). Univariate regression models, including crude univariate models (Model 1) and univariate models adjusted for meteorological covariates (Model 2), were built to analyze how individual environmental factors are linked to RSV transmission in Japan. Multivariate regression models (Model 3) were developed using stepwise methods (Supplementary Data, page 3) for both meteorological factors and air pollutants separately to improve predictive performance while avoiding overfitting in order to better understand the complex associations between environmental factors and RSV infections in Japan. We used directed acyclic graphs (DAGs) to clarify the presumed associations among variables based on existing evidence (Supplementary Figures 1–2) [29–31]. Weekly mean average temperature, wind speed, relative humidity, weekly total precipitation, weekly temperature range, weekly number of cold days (days with average temperatures <0°C), and weekly number of hot days (days with average temperatures >30°C) were included in our multivariate meteorological model as exposures, according to existing evidence [8, 32, 33]. Weekly mean visibility and sea level pressure were controlled as covariates as they exhibit correlations with both exposures and RSV cases (Supplementary Figure 3). Concurrently, weekly mean concentrations of $NO_{2}$, $SO_{2}$, CO, and $PM_{2.5}$ were included in our multivariate air pollutant model as exposures. In the air pollutant model, we controlled for weekly mean sea level pressure, relative humidity, wind speed, and average temperature as covariates (Supplementary Figure 3). We compared our proposed model with commonly used statistical models to evaluate its performance (Supplementary Data, pages 3 and 4). Details about model specifications are provided in the “Statistical Modeling” section. Given the substantial variation in temperature conditions across Japan, subgroup analyses were conducted based on geographical regions to assess univariate associations between weekly average temperature and RSV infections. We categorized the 47 Japanese regions into 3 groups based on the latitudes of 30° and 35°.

The third phase evaluates which environmental factors could contribute to predicting RSV transmission. We employed a mixed-effects machine learning (ML) model, Gaussian Process Boosting (GPBoost) [34], which combines tree-boosting and Gaussian Process (GP) modeling, to predict weekly RSV cases. Linear regression (LR) [35], random forest (RF) [36], and eXtreme Gradient Boosting (XGBoost) [37] were used as baselines. A total of 26 features were utilized, including historical environmental data and RSV cases from 1 week prior, as well as spatial and temporal indicators such as longitude, latitude, week, and month. The data set was divided into training (data from 2013–2018) and testing (data from 2019) subsets. Grid search and 5-fold cross-validation on the training data were applied to fine-tune hyperparameters in these models (Supplementary Data, page 4). We assessed the performance of each model on the testing data using 3 metrics, including mean absolute error (MAE), root mean square error (RMSE), and $R^{2}$ [38], to determine the optimal model. Feature importance was calculated using SHapley Additive exPlanations (SHAP) [39], a game-theoretic approach that interprets how ML models make predictions.

All quantitative analyses were conducted using R, version 4.2.1. The *dtwclust* [40], *dlnm* [41], *mgcv* [42], and *sp* [43] packages were used to develop the regression model. The *xgboost* [44], *tidymodels* [45], *gpboost* [46], and *SHAPforxgboost* [47] packages were used to perform prediction analysis. The relative risk (RR) and 95% CI were calculated, and all tests were 2-sided, with α = .05.

### Statistical Modeling

The HSDLNM contains the Distributed Lag Nonlinear Model (DLNM), Markov Random Field (MRF), and random intercept term. The DLNM allows for flexibly assessing the associations between the outcome and exposures that vary over time while accounting for both delayed (lagged) effects and nonlinear relationships [48]. The MRF is a graphical model representing the dependencies of random variables defined on a spatial domain [49]. It excels at handling spatial heterogeneity in area-based data [18]. Additionally, the random intercept term allows the model to accommodate various time-series patterns identified by clustering in phase I, accounting for unobserved heterogeneity between patterns. $Y_{\mathrm{itkc}}$ is the number of RSV cases at week t in year k in prefecture i and time-series cluster c. We assumed that $Y_{\mathrm{itkc}}$ follows a Poisson distribution, for example, $Y_{\mathrm{itkc}}∼\mathrm{Pois}(\lambda_{\mathrm{itkc}})$, where $\lambda_{\mathrm{itkc}}$ is the expected mean of $Y_{\mathrm{itkc}}$. In our models, we considered an extended version of Poisson regression, quasi-Poisson regression, to solve the overdispersion issue. Models 1, 2, and 3, described above, are set out in equations (1)–(3).


(1) $$\begin{array}{rl} \log(\lambda_{\mathrm{itkc}}) & =\alpha +S_{\mathrm{cluster}}(c)+f(\mathrm{factor},\mathrm{lag})+S_{\mathrm{spatial}}(i)\\ & \;+S(\mathrm{tim}e_{t}|\mathrm{yea}r_{k})+\varepsilon ,\varepsilon \in N(0,\sigma^{2}) \end{array}$$

(2) $$\begin{array}{rl} \log(\lambda_{\mathrm{itkc}}) & =\alpha +S_{\mathrm{cluster}}(c)+f(\mathrm{factor},\mathrm{lag})+f(\mathrm{covariates},\mathrm{lag})\\ & \;+S_{\mathrm{spatial}}(i)+S(\mathrm{tim}e_{t}|\mathrm{yea}r_{k})+\varepsilon ,\varepsilon \in N(0,\sigma^{2}) \end{array}$$

(3) $$\begin{array}{rl} \log(\lambda_{\mathrm{itkc}}) & =\alpha +S_{\mathrm{cluster}}(c)+f(\mathrm{factors},\mathrm{lag})+f(\mathrm{covariates},\mathrm{lag})\\ & \;+S_{\mathrm{spatial}}(i)+S(\mathrm{tim}e_{t}|\mathrm{yea}r_{k})+\varepsilon ,\varepsilon \in N(0,\sigma^{2}) \end{array}$$

Here, α is the overall mean, and the random intercept term $S_{\mathrm{cluster}}(c)$ allows different time-series patterns to adjust the baselines flexibly and control for variations between groups. f(factor,lag) measures the lagged effect of individual environmental factors on RSV cases up to 3 weeks. The cross-basis functions were composed of a linear or piecewise linear exposure–response relationship and a polynomial regression in a lag–response relationship with 3 degrees of freedom (df). f(covariates,lag) are composed of cross-basis functions of P-splines with 5 df for both exposure–response and lag–response relationships. The hierarchical nonlinear $S(\mathrm{tim}e_{t}|\mathrm{yea}r_{k})$ is a smooth term of thin plate regression splines to control for seasonality and trend. The spatial function $S_{\mathrm{spatial}}(i)$ is an MRF with a conditional autoregressive prior, which takes into account the spatial structure of 47 prefectures. Specifically, we used the first-order neighborhood structure [50] to represent spatial structure, where prefectures that share a boundary are considered neighbors, with an area conditionally independent (given its neighbors) of any non-neighboring areas.

### Sensitivity Analyses

Multiple sensitivity analyses were performed to examine the robustness of our study. For association analysis, we first conducted analyses extending the lag period of environmental variables to 5 weeks to observe the lag–response structures. Second, we removed covariates from multivariate models and examined the effects on primary effect estimates. For the predictive analysis, we randomly split the data set into training (80%) and testing (20%) data, stratified by year. We assessed model performance on the randomly split testing data set to evaluate the effectiveness of the GPBoost model.

## RESULTS

### Data Characteristics

In our study, a total of 816 380 RSV cases were reported across 47 prefectures from 2013 to 2019 in Japan. The mean (SD) of the weekly number of lab-confirmed RSV-positive cases was 47.6 (73.3). The characteristics of environmental conditions in Japan are summarized in Table 1. No significant spatial patterns were found in the annually aggregated RSV cases from 2013 to 2019 (Supplementary Table 2, Supplementary Figure 4), indicating a random distribution of RSV infections across Japan over the study period. The time-series decomposition result (Supplementary Figure 5) showed that the trend component is dominant compared with the seasonal component, indicating that the variation in the trend component is large. The trend pattern of RSV cases revealed an upsurge of RSV infections in 2017 and 2019. Additionally, the seasonal pattern of RSV cases revealed a peak in the autumn and winter seasons. Four distinct time-series patterns were found across 47 prefectures in Japan (Supplementary Figure 6).

**Table 1.** Summary Statistics for Weather Conditions and RSV Activity Nationwide in Japan Between 2013 and 2019

…	Mean (SD)	Min	25^th^	50^th^	75^th^	Max	IQR
RSV activity	…	…	…	…	…	…	…
No. RSV cases	47.6 (73.3)	0	7	22	57	889	50
Meteorological (weekly)	…	…	…	…	…	…	…
Average relative humidity	69.9 (9.4)	32.2	63.7	70.5	76.6	96.6	12.9
Average wind speed	10.4 (3.4)	2.8	8	10	10.2	42.5	4.2
Total precipitation	32.7 (43.4)	0	4.6	19.1	43.4	564.5	38.8
Mean temperature	15.8 (8.5)	−6.9	8.4	16.4	23	32.3	14.6
Temperature range	14.1 (3.5)	2.3	11.6	13.7	16.2	27.9	4.6
Air pollutants (weekly, 99th percentile)	…	…	…	…	…	…	…
PM2.5	12.4 (5.5)	1.2	8.7	11.5	15.1	86.6	6.4
NO2	37.7 (57.5)	4.4	21.9	30.4	42.4	1436.4	20.5
SO2	14.4 (32.9)	0	6.4	9.9	15.6	723.6	9.2
CO	4.3 (1.4)	0	3.4	4.2	5	13.8	1.6

Abbreviations: IQR, interquartile range; RSV, respiratory syncytial virus.

### Associations Between Meteorological Factors and RSV Activity

Correlation analysis showed significant correlations between all meteorological variables and weekly confirmed RSV cases, except for wind speed (Supplementary Figure 3). Preliminary trends in associations between meteorological factors and RSV activity are presented in Supplementary Figures 7 and 8. The stepwise model selection results for meteorological factors are presented in Supplementary Table 3. The proposed HSDLNM model outperformed commonly applied regression models, achieving the highest $R^{2}\;$(Supplementary Table 4).

**Figure 2.**
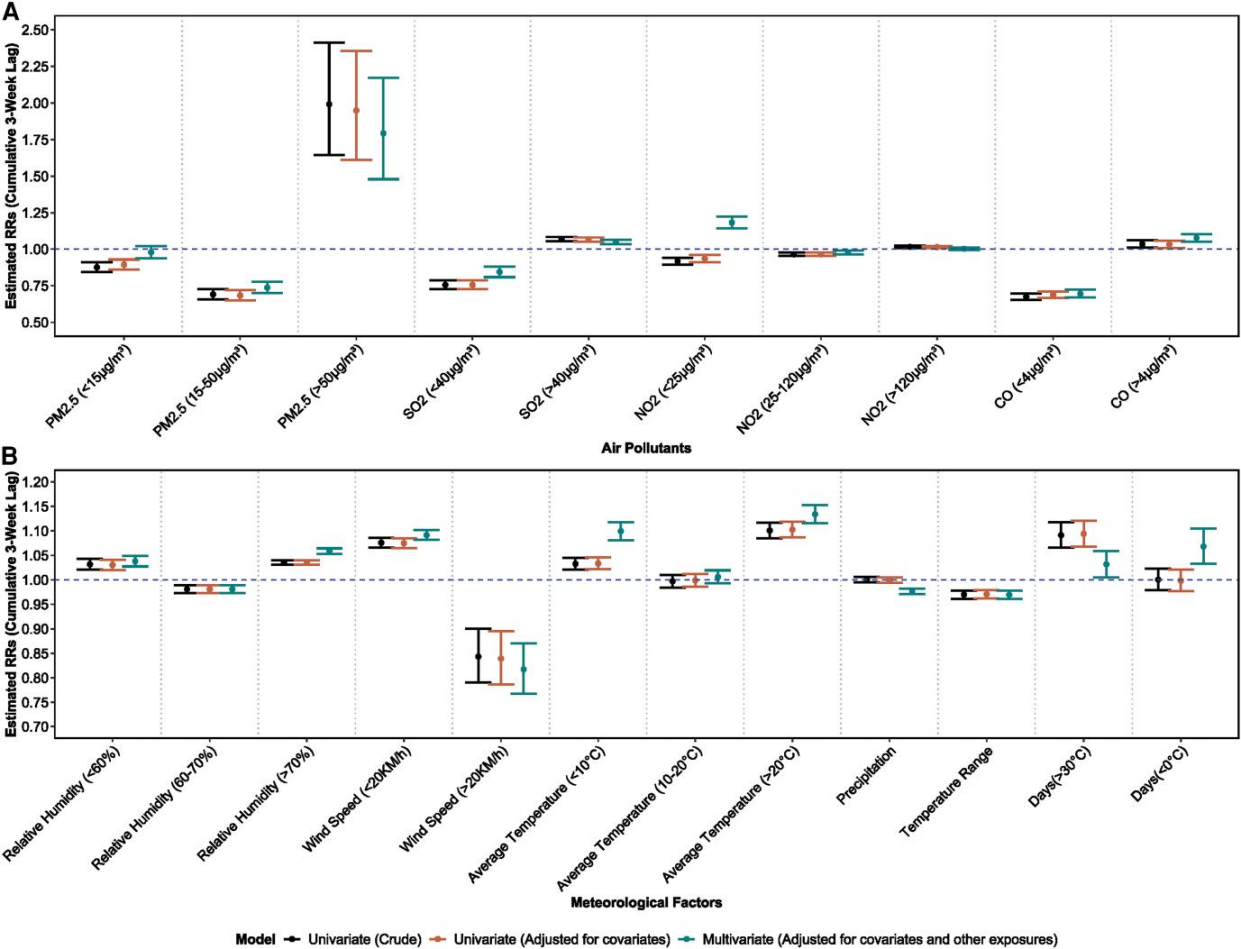
Associations between environmental conditions and RSV activity at a cumulative 3-week lag in Japan, 2013–2019. *A*, Associations of short-term exposures to PM2.5, SO2, NO2, and CO. *B*, Associations of meteorological factors, including relative humidity, wind speed, average temperature, total precipitation, temperature range, days >30°C, and days <0°C. Abbreviations: RR, relative risk; RSV, respiratory syncytial virus.

The 3-week pooled RR estimates of associations between meteorological factors and RSV cases are presented in Figure 2. Weekly mean relative humidity, wind speed, and average temperature showed varying associations with RSV cases under different thresholds but consistent trends in both crude and adjusted univariate models and the multivariate model. According to the multivariate regression model, after controlling for other exposures and covariates, we observed that the RRs of RSV infections for every 1% increase in the relative humidity were 1.04 (relative humidity: <60%; 95% CI, 1.03–1.05), 0.98 (relative humidity: 60%–70%; 95% CI, 0.97–0.99), and 1.06 (relative humidity: >70%; 95% CI, 1.05–1.06). Meanwhile, a 1°C increase in average temperature was associated with an elevated RR of RSV infections when it was <10°C (RR, 1.10; 95% CI, 1.08–1.12) and >20°C (RR, 1.13; 95% CI, 1.12–1.15) in the multivariate regression model. Wind speed had 2 distinct effects on RSV infections. Every 1-km/h increase in wind speed when it was <20 km/h was associated with a 9% higher risk of RSV infections (RR, 1.09; 95% CI, 1.08–1.1) and an 18% lower risk of RSV infections when it was >20 km/h (RR, 0.82; 95% CI, 0.77–0.87), according to the multivariate regression model. The weekly temperature range showed consistent negative associations with RSV infections in all 3 regression models, with a 1°C increase associated with a 3% lower risk of RSV infections (RR, 0.97; 95% CI, 0.96–0.98) in the multivariate model, while controlling for other exposures and covariates. No significant associations were found between weekly total precipitation and RSV infections in crude and adjusted univariate regression models. After controlling for other exposures and covariates, precipitation showed negative associations with RSV infections in the multivariate regression model, with a 1-mm increase associated with a 2% lower risk of RSV infections (RR, 0.98; 95% CI, 0.97–0.98). Extreme temperatures, both the weekly number of cold days (days with average temperatures <0°C) and the weekly number of hot days (days with average temperatures >30°C), exhibited positive associations with RSV infections in the multivariate regression model. Every 1-day increase in cold days was associated with a 7% higher risk of RSV infections (RR, 1.07; 95% CI, 1.03–1.10), and every 1-day increase in hot days was associated with a 3% higher risk of RSV infections (RR, 1.03; 95% CI, 1.01–1.06) in the multivariate regression model. No significant associations were found between cold days and RSV infections in crude and adjusted univariate regression models.

**Figure 3.**
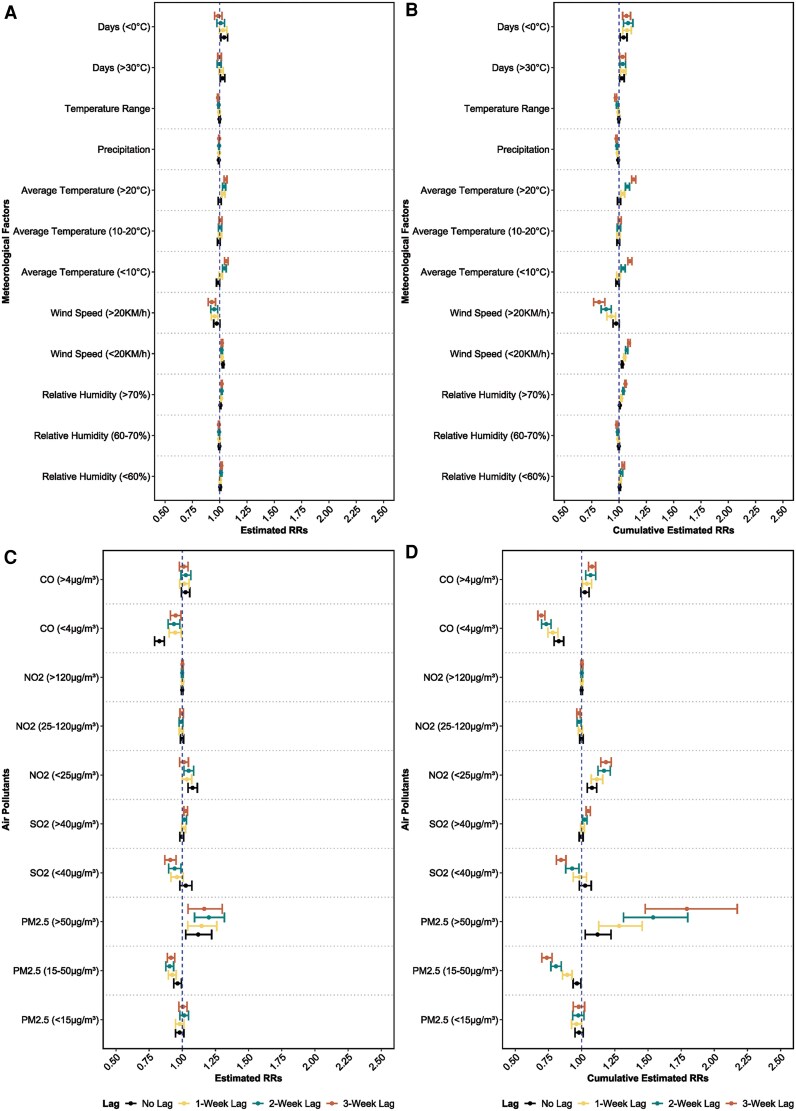
Results of multivariate associations between environmental conditions and RSV activity at different week lags in Japan, 2013–2019. *A*, Associations between meteorological factors and RSV activity at different single weeks of lag in the multivariate model, from lag 0 to lag 3. *B*, Associations between meteorological factors and RSV activity at different cumulative weeks of lag in the multivariate model, from lag 00 to lag 03. *C*, Associations between air pollutants and RSV activity at different single weeks of lag in the multivariate model, from lag 0 to lag 3. *D*, Associations between air pollutants and RSV activity at different cumulative weeks of lag in the multivariate model, from lag 00 to lag 03. Abbreviations: RR, relative risk; RSV, respiratory syncytial virus.

Detailed multivariate associations between meteorological factors and RSV cases at different time lags up to 3 weeks are presented in Figure 3. All meteorological factors, except for average temperature, showed stable relationships with RSV infections. Average temperatures <10°C exhibited unstable relationships with RSV infections at different time lags, especially in the univariate models (Supplementary Figures 9 and 10). The latitude-stratified results of univariate (adjusted) associations between average temperature and RSV infections, adjusting for covariates, are presented in Supplementary Figure 11. Below 30° latitude, the 3-week pooled RRs of RSV infections for every 1°C increase in average temperature <20°C and average temperature >20°C were 0.79 (95% CI, 0.68–0.92) and 1.15 (95% CI, 1.06–1.25), respectively. Between 30° and 35° latitude, every 1°C increase in average temperature (>20°C) was associated with an increased risk of RSV infections, with a 3-week pooled RR of 1.15 (95% CI, 1.13–1.18). At latitudes above 35°, the 3-week pooled RR of RSV infections for every 1°C increase in average temperature 10°C–20°C and average temperature >20°C were 1.05 (95% CI, 1.03–1.07) and 1.10 (95% CI, 1.08–1.13), respectively.

### Associations Between Air Pollutants and RSV Activity

A correlation analysis showed significant correlations between all air pollutants and weekly confirmed RSV cases (Supplementary Figure 6). Preliminary trends in associations between air pollutants and RSV activity are presented in Supplementary Figures 12 and 13. The stepwise model selection results for air pollutants are presented in Supplementary Table 5.

Associations between air pollutants and RSV cases are presented in Figure 2. Weekly mean concentrations of $NO_{2}$, $SO_{2}$, CO, and $PM_{2.5}$ showed varying associations with RSV cases under different thresholds. Univariate associations are presented in Supplementary Figures 14 and 15. In the adjusted univariate regression models, the 3-week pooled RRs of RSV infections for every 5- $\mu g/m^{3}$ increase in concentrations of $SO_{2}$ (>40 $\mu g/m^{3}$) and $PM_{2.5}$ (>50 $\mu g/m^{3}$) were 1.07 (95% CI, 1.05–1.08) and 1.95 (95% CI, 1.61–2.36), respectively. After controlling for other air pollutants in the multivariate regression model (Figure 3), the 3-week cumulative RRs of RSV infections for every 5- $\mu g/m^{3}$ increase in concentrations of $NO_{2}$ (<25 $\mu g/m^{3}$), $SO_{2}$ (>40 $\;\mu g/m^{3}$), and $PM_{2.5}$ (>50 $\mu g/m^{3}$) and for every 1- $\mu g/m^{3}$ increase in concentrations of CO (>4 $\mu g/m^{3}$) were 1.18 (95% CI, 1.14–1.22), 1.05 (95% CI, 1.03–1.06), 1.79 (95% CI, 1.48–2.17), and 1.08 (95% CI, 1.05–1.10), respectively.

### Interpretable Machine Learning Predictions

**Figure 4.**
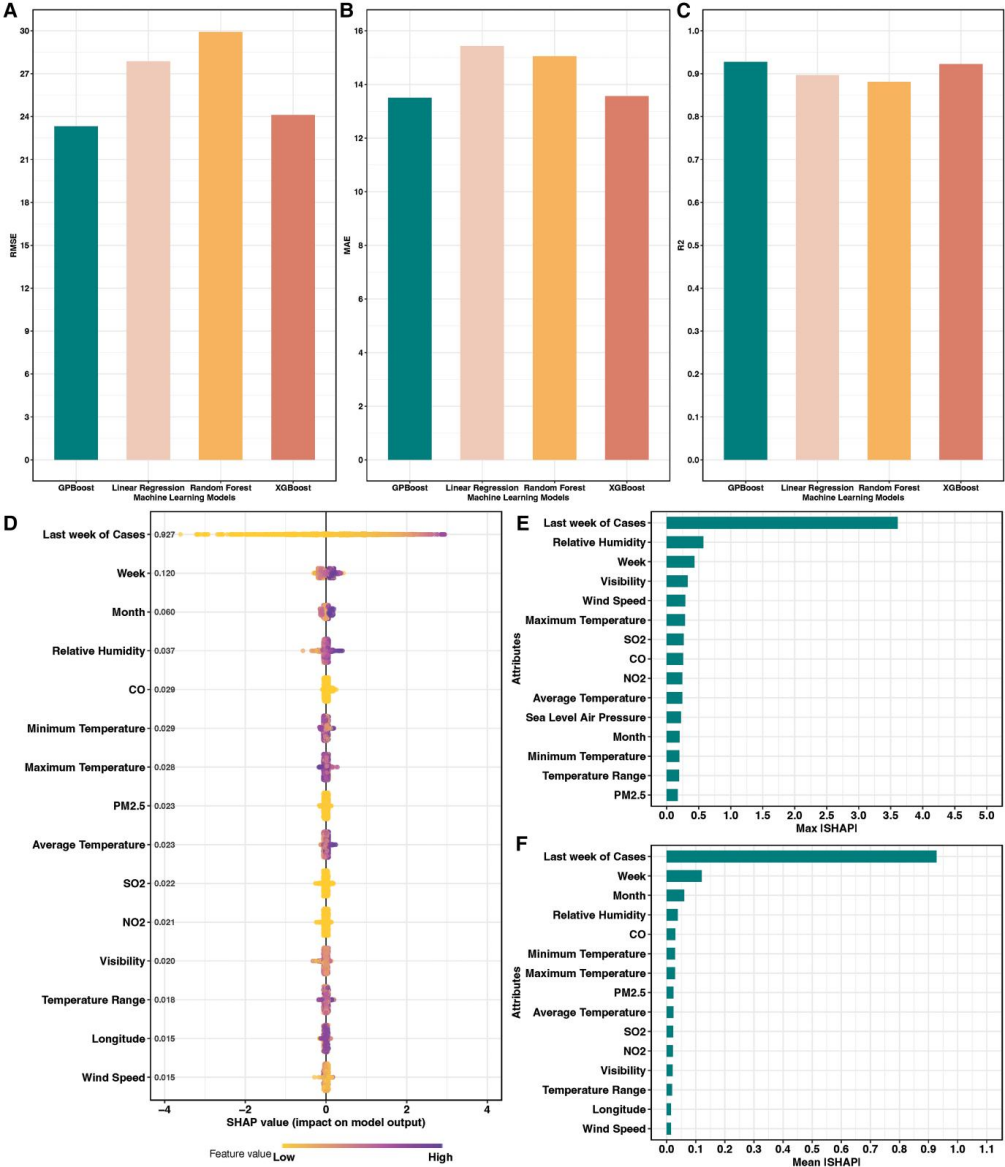
Interpretable machine learning performance and results. *A*, Plot of RMSE for 4 different machine learning models on the testing data set. *B*, Plot of MAE for 4 different machine learning models on the testing data set. *C*, Plot of R² for 4 different machine learning models on the testing data set. *D*, Summary plot of SHAP values of the top 15 features. *E*, Plot of maximum absolute SHAP values of the top 15 features. *F*, Plot of mean absolute SHAP values of the top 15 features. Abbreviations: ISHAPI, absolute SHAP value; MAE, mean absolute error; RMSE, root mean square error; RR, relative risk; SHAP, SHapley Additive exPlanations.

The summarized performance of 4 machine learning models on the testing data set is shown in Figure 4. The GPBoost model outperformed 3 baseline models, with the lowest RMSE (23.32), lowest MAE (13.5), and highest R² (0.93). The SHAP values presented in Figure 4 were estimated from the GPBoost model to understand the impact of individual input variables on the model's predictions. We ranked the importance of variables for predicting RSV cases according to mean absolute SHAP values. The results indicated that the number of RSV cases in the previous week was the most significant predictor of the following week's RSV case counts. Other important predictors included temporal features (month, week) and environmental conditions, including weekly average relative humidity, weekly mean concentrations of CO, weekly minimum, maximum, and average temperature, and weekly mean concentrations of $PM_{2.5}$, $NO_{2}$, and $SO_{2}$ in the previous week.

### Sensitivity Analyses

Sensitivity analyses (Supplementary Figures 16–19) validated the robustness of our findings. In the multivariate meteorological model, our results remained consistent when models were individually adjusted for weekly mean visibility or weekly sea level pressure as covariates, as well as when no covariates were included. Similarly, results remained robust when relative humidity, average temperature, and wind speed were separately removed from the multivariate air pollution model. Furthermore, our results were stable when we extended the lag period by 4 and 5 weeks. Additionally, on the randomly split testing data set, the GPBoost model outperformed other baselines, achieving the lowest MAE and RMSE and the highest R².

## DISCUSSION

Our nationwide study of Japan has identified complex associations between environmental factors, both meteorological factors and air pollutants, and RSV activity in the country from 2013 to 2019. All meteorological factors showed significant associations with RSV activity in the multivariate model. Specifically, higher relative humidity (>70%), higher average temperature (>20°C), higher wind speed (<20 km/h), and extreme cold and hot days are linked to higher risks of RSV infections. Additionally, lower precipitation and temperature fluctuations per week are associated with higher risks of RSV infection. All air pollutants showed significant association with RSV activity in the multivariate model. Except for $NO_{2}$, increased exposure to other air pollutants, viz., $SO_{2}$, CO, and $PM_{2.5}$, was associated with a higher risk of RSV infection. To our knowledge, this is the first study to examine the association between both air pollutants and meteorological factors and RSV activity in Japan.

Our findings suggest that both lower (<10°C) and higher (>20°C) temperatures are associated with enhanced RSV transmission, consistent with prior studies [32, 51]. Laboratory studies have explored the relationship between temperature and viral infectivity. Experiments with mouse hepatitis virus (MHV) found that under consistent humidity levels, altering the temperature to either 30°C or 10°C significantly preserved the infectivity of MHV, when compared with a control condition of 21°C [52]. Similar findings were observed for the severe acute respiratory syndrome virus, noting its prolonged survival at 4°C for up to 14 days in sewage, in contrast to a mere 2-day survival at 20°C [53]. Additionally, previous research noted that in tropical settings, high humidity is generally accompanied by high temperature, which appears to influence human behaviors, particularly the preference for indoor activities in air conditioned environments [54]. Such indoor settings, characterized by recirculated air and closer interpersonal interactions, potentially enhance person-to-person virus transmission [55].

In our study, a positive association between relative humidity (>70%) and RSV transmission was observed. Similar patterns have also been shown in previous research in other countries [32, 56–58 ]. High relative humidity may create conditions conducive to RSV viability and transmission, enhancing the virus's stability within large-particle aerosols and facilitating its year-round spread. Research by Rechsteiner et al. [59] supported this hypothesis, revealing that RSV inactivation rates decline markedly at 90% relative humidity, especially within the initial 16 minutes postaerosolization, compared with significantly higher inactivation rates observed at 60% humidity. Similarly, research by Oswin et al. [52] demonstrated an escalation in the infectivity of MHV with increased relative humidity levels—70% and 90%—in comparison with lower humidity conditions (30%) under constant temperature settings. These findings further disclose the critical influence of relative humidity on the transmission dynamics of RSV.

Several analyses have reported the effects of short-term exposure to air pollutants on respiratory infections, especially RSV. Our study demonstrated statistically significant associations of reported RSV cases with $NO_{2}$, $SO_{2}$, CO, and $PM_{2.5}$, in general agreement with previous studies in other countries [9, 10, 60]. Additionally, we found that CO, $PM_{2.5}$, and $SO_{2}$ are important indicators when forecasting RSV infections. The mechanisms of how high-level concentrations of air pollutants facilitate respiratory infections help explain our findings. Exposure to high levels of $PM_{2.5}$ could result in increased vulnerability to RSV infections. Higher $PM_{2.5}$ concentrations would impair lung function by increasing bronchial hyperresponsiveness and weakening the antimicrobial defense system of the lungs, and also decreasing lung mRNA levels of antioxidant enzymes [6, 61]. Exposure to high levels of CO could induce pulmonary edema, immune cell infiltration, and increased COHb, which result in increased risks of respiratory infections [62]. Exposure to high-level concentrations of $SO_{2}$ can cause strong irritation of the respiratory mucosa, mucosal congestion, edema, and inflammatory exudation, resulting in increased airway secretion discharge and destruction of the mucosal structure, increasing vulnerability to RSV infections [63].

Our innovative analytical framework, adept at integrating spatial and temporal variations, has the capacity to accurately map the spatiotemporal patterns of RSV cases. Our analysis emphasizes the importance of designing models based on the characteristics of the study data set. First, the proposed HSDLNM approach showed significant advancements over conventional regression models in delineating the intricate relationships between environmental variables and RSV activity. Unlike traditional linear models, it can capture the complexity of the time-dependent relationships prevalent in epidemiological data, avoiding oversimplified interpretations or skewed conclusions about such relationships. The MRF function is adept at processing area-based spatial data, tackling the spatial heterogeneity in multiregion disease data. Our comparative regression analysis underscored the advantages of the HSDLNM approach in fitting spatiotemporal data. Second, we utilized the mixed-effects ML model, GPBoost, to predict weekly RSV infections. The GPBoost model better handles multiregional prediction tasks. We speculate that it does so by capturing the correlations between areas, in contrast to traditional ML models such as XGBoost. The GPBoost model surpassed other baseline models in forecasting weekly RSV cases in Japan in the testing data set. Our sensitivity analyses further confirm the robustness of our proposed analysis framework, underscoring the reliability of our framework for assessing the impact of environmental factors on RSV transmission.

In summary, our study introduced a novel analytical framework to help elucidate the spatiotemporal patterns of RSV transmission and how environmental variables are associated with RSV infections in Japan. However, this study has several limitations. First, our analysis was limited by the absence of age-specific disease data and insufficient information on catchment populations, which prevented us from calculating incidence rates/attack rates. Consequently, our study relied on the examination of raw case counts rather than employing age-standardized incidence rates, which could have provided a more comprehensive understanding of RSV transmission dynamics. Second, although circulating viral genotypes may influence infectivity and viral transmission [64], our model falls short in incorporating RSV genotype information due to the unavailability of genotype-specific data (ie, RSV-A and RSV-B). Future research could explore incorporating genotype data into spatiotemporal analyses across different parts of the world. Third, our study was conducted exclusively using data from the prepandemic period (2013–2019). The coronavirus disease 2019 pandemic significantly disrupted typical circulation patterns and seasonal dynamics of many respiratory viruses, including RSV, which has now largely returned to prepandemic patterns [65]. Therefore, the generalizability of our model to the immediate postpandemic data (2021–2023) is uncertain, as altered data patterns may reduce the model's predictive accuracy. Fourth, although our model integrated spatial relationships to improve model accuracy, it fell short of precisely delineating how environmental impacts on RSV vary across different provinces and regions. In our subgroup analysis, we built latitude-stratified models to explain the unstable relationships between average temperature and RSV infections. Future studies should build individual models for each prefecture in Japan to capture regional variations more accurately. Fifth, this study elucidated the lagged effects of environmental factors on RSV activity in Japan. However, this analysis did not establish any causal relationship between environmental factors and RSV activity. Lastly, the lack of availability of suitable external data sets precluded the validation of our framework's applicability to other contexts. Despite sensitivity analyses stating the robustness of our framework, its generalizability to regions outside Japan remains to be confirmed.

## CONCLUSIONS

Our proposed framework captured the intricate spatiotemporal dynamics of RSV transmission, enabling precise identification of the environmental factors significantly associated with RSV infections. Spatial-temporal features and environmental factors showed potential in forecasting RSV activities in Japan, offering insights for health service planning and prevention. Consequently, our study contributes to the enhancement of future health service planning, enabling more effective prevention strategies and interventions aimed at reducing RSV infections and outbreak severity.

## Supplementary Material

ofaf392_Supplementary_Data

## Supplementary Data

Supplementary materials are available at *Open Forum Infectious Diseases*  online. Consisting of data provided by the authors to benefit the reader, the posted materials are not copyedited and are the sole responsibility of the authors, so questions or comments should be addressed to the corresponding author.
