# Supplementary material for: Associations Between Environmental Conditions and Infection With Respiratory Syncytial Virus in Japan: A Spatiotemporal Analysis
Source: Open Forum Infect Dis. 2025 Aug 13;12(8):ofaf392. doi: 10.1093/ofid/ofaf392 (PMC12345628; doi:10.1093/ofid/ofaf392)
Supplement: ofaf392_Supplementary_Data [file ofaf392_supplementary_data.docx]

**Content**

**Data Imputation**

**Regression Analysis**

Basis Function Selection Procedures

Variable Selection Procedures

Comparative Analysis for Regression Models

**Forecasting Analysis**

Model Training

Model Testing

**Tables**

Table S1. Summary statistics for air pollutants in nationwide Japan between 2013 and 2019

Table S2. Spatial autocorrelation (Global Moran’s I statistic) for RSV cases in Japan between 2013 and 2019

Table S3. Table of stepwise model selection results for meteorological factors

Table S4. Summary of regression model performance

Table S5. Table of stepwise model selection results for air pollutants

**Figures**

Figure S1. Directed acyclic graph of relationships among meteorological factors and RSV infections

Figure S2. Directed acyclic graph of relationships among air pollutants and RSV infections

Figure S3. Spearman’s rank correlation matrix of weather conditions and respiratory syncytial virus activity in Japan

Figure S4. Maps of the geographic distribution of total RSV cases in Japan from 2013 to 2019

Figure S5. Decomposition of weekly RSV cases in Japan from 2013 to 2019 using STL

Figure S6. A time-series plot of distinct RSV infection patterns

Figure S7. Trends in associations between meteorological factors and respiratory syncytial virus activity at different cumulative lags up to 3 weeks

Figure S8. Plots of linear association between meteorological factors and respiratory syncytial virus activity up to 3 weeks of lag in the adjusted univariate model

Figure S9. Plots of association between meteorological factors and respiratory syncytial virus activity up to 3 weeks of lag in the crude univariate model

Figure S10. Plots of association between meteorological factors and respiratory syncytial virus activity up to 3 weeks of lag in the adjusted univariate model

Figure S11. Plots of subgroup analysis results of average temperature models

Figure S12. Trends in associations between air pollutants and respiratory syncytial virus activity at different cumulative lags up to 3 weeks

Figure S13. Association between air pollutants and respiratory syncytial virus activity up to 3 weeks of lag based on WHO air quality guidelines (2005)

Figure S14. Plots of association between air pollutants and respiratory syncytial virus activity up to 3 weeks of lag in the crude univariate model

Figure S15. Plots of association between air pollutants and respiratory syncytial virus activity up to 3 weeks of lag in the adjusted univariate model

Figure S16. Plots of model sensitivity for changing covariates in meteorological models, up to 3 weeks of lag

Figure S17. Plots of model sensitivity for changing lag time in meteorological models

Figure S18. Plots of model sensitivity for changing covariates in air pollutant models, up to 3 weeks of lag

Figure S19. Plots of model sensitivity for changing lag time in air pollutant models

**Data Imputation**

Our dataset contained missing values for three variables: weekly maximum temperature, weekly minimum temperature, and weekly mean CO concentration. To address this, we built machine learning models to help impute the missing values.

First, we excluded all missing values from the original dataset. Then, we split the dataset into training data (80%) and testing data (20%). We applied three separate XGBoost models to predict the weekly maximum temperature, weekly minimum temperature, and weekly mean CO concentration, respectively, using other environmental factors as features. For each model, we employed 5-fold cross-validation and grid search to identify the optimal hyperparameter combinations. After determining the best combination, we trained the optimal model on the entire training dataset. We then evaluated its performance on the testing data using R-squared ($R^{2}$). Finally, the optimal model was used to predict the missing values.

**Regression Analysis**

**Basis Function Selection Procedures**

First, we applied natural cubic splines as basis functions to identify the trend of exposure-response relationships between environmental variables and RSV cases up to 3 weeks. Based on these trends, we defined our exposure-response relationships for the main analysis. For trends that approximate linearity, we used linear models as base functions. For nonlinear relationships, we employed piecewise linear models to improve interpretation. For meteorological factors, we selected candidate thresholds for piecewise linear models based on the turning points of nonlinear trends and defined the thresholds based on the goodness of fit of candidate models. For air pollutants, we initially set thresholds according to the World Health Organization’s (WHO) air quality guidelines (2005).^1^ For $\mathrm{PM}_{2.5}$ and $\mathrm{NO}_{2}$, we further refined these thresholds. We defined additional thresholds for $\mathrm{PM}_{2.5}$ above 15 $\mathrm{ug}/m^{3}$, e.g., $\mathrm{PM}_{2.5} ($15-50 $\mathrm{ug}/m^{3}$) and $\mathrm{PM}_{2.5} ($>50 $\mathrm{ug}/m^{3}$), since average concentrations above 50 $\mathrm{ug}/m^{3}$ are considered poor air quality and pose serious health risks according to the Environmental Protection Authority (EPA).^2^ For $\mathrm{NO}_{2}$, we set thresholds above 25 $\mathrm{ug}/m^{3}$, e.g., $\mathrm{NO}_{2}$(25-120 $\mathrm{ug}/m^{3}$) and $\mathrm{NO}_{2}$(>120 $\mathrm{ug}/m^{3}$), noting that short-term exposure to concentrations above 120 µg/m³ has been reported to have negative effects on ecosystems and respiratory systems.^3^

**Variable Selection Procedures**

In our dataset, we have weekly metrics of environmental factors that are highly correlated with each other, such as average temperature, maximum temperature, and minimum temperature. To avoid multicollinearity and improve the fitting ability of our regression models, we compared the goodness of fit of different variables in the adjusted univariate models and used a stepwise method to select variables for incorporation into the multivariate models (**Table S3-S4**).

**Comparative Analysis for Regression Models**

Comparative analysis was performed on five different types of regression models to evaluate the performance of our proposed model, including:

1. baseline models: linear model (LM) and generalized linear model (GLM),
2. models that deal with highly correlated predictors: weighted quantile sum model (WQS),
3. models that incorporate temporal effect: distributed lag nonlinear model (DLNM),
4. models that incorporate spatial variation: generalized additive model (GAM),
5. models that incorporate both spatial variation and temporal effects: DLNM with different spatial smooth terms,
6. our proposed model: HSDLNM

We evaluated the model performance of the above models across five metrics, including adjusted $R^{2}$, mean absolute error (MAE), root mean square error (RMSE), mean square error (MSE), and explained deviance (%) (**Table S5**).

**Forecasting Analysis**

**Model Training**

In this study, we applied a 5-fold cross-validation method, dividing the training data into five equal parts. Random search (n = 50) was used to explore potential hyperparameter combinations for model tuning. The training process is detailed as follows.

For each hyperparameter combination, the model was fitted five times. In each iteration, one subset of the data serves as the validation set, while the remaining four subsets form the new training set. The performance of each model is evaluated using three metrics: MAE, RMSE, and $R^{2}$. After five iterations, the performance metrics from each validation set are averaged to produce the final score for that model. Once the random search is complete, the hyperparameter combination with the lowest average validation score (MAE and RMSE) is selected as optimal. After determining the best combination, we trained the optimal model on the entire training dataset.

**Model Testing**

After the training process, we identified the best parameter sets for the XGBoost, Random Forest, and GPBoost models. These models were then fitted to the testing dataset and evaluated using MAE, RMSE, and $R^{2}$. The model with the lowest values for MAE and RMSE, and the highest value of $R^{2}$, was selected as the optimal model.

**Table S1. Summary statistics for air pollutants in nationwide Japan between 2013 and 2019**

|  | **Mean (SD)** | **Min** | **25th** | **50th** | **75th** | **Max** | **IQR** |
| --- | --- | --- | --- | --- | --- | --- | --- |
| **Air Pollutants (Weekly)** |  |  |  |  |  |  |  |
| PM2.5 | 17 (59.9) | 1.2 | 8.7 | 11.6 | 15.2 | 3953.5 | 6.5 |
| NO2 | 72.5 (338.1) | 4.4 | 21.9 | 31.1 | 43.5 | 9174.5 | 21.6 |
| SO2 | 43 (295.7) | 0 | 6.4 | 9.9 | 15.6 | 9176.9 | 9.2 |
| CO | 20.9 (245.3) | 0 | 3.4 | 4.2 | 5 | 6455 | 1.6 |
| **Air Pollutants (Weekly, 99 percentile)** |  |  |  |  |  |  |  |
| PM2.5 | 12.4 (5.5) | 1.2 | 8.7 | 11.5 | 15.1 | 86.6 | 6.4 |
| NO2 | 37.7 (57.5) | 4.4 | 21.9 | 30.4 | 42.4 | 1436.4 | 20.5 |
| SO2 | 14.4 (32.9) | 0 | 6.4 | 9.9 | 15.6 | 723.6 | 9.2 |

**Table S2.** **Spatial autocorrelation (Global Moran’s I statistic) for RSV cases in Japan between 2013 and 2019**

| **Year** | **Moran’s I statistic** | **p-value** | **Spatial Pattern** |
| --- | --- | --- | --- |
| 2013 | -0.041 | 0.598 | random |
| 2014 | -0.071 | 0.734 | random |
| 2015 | -0.066 | 0.708 | random |
| 2016 | -0.027 | 0.512 | random |
| 2017 | -0.031 | 0.526 | random |
| 2018 | -0.04 | 0.573 | random |
| 2019 | -0.037 | 0.513 | random |

**Table S3.** **Table of stepwise model selection results for meteorological factors**

| **Stepwise Selection** | **R2** | **Explained Deviance (%)** |
| --- | --- | --- |
| Linear Relative Humidity | 0.551 | 0.630 |
| Linear TAVG | 0.548 | 0.627 |
| Linear Wind Speed | 0.556 | 0.631 |
| Linear Precipitation | 0.547 | 0.626 |
| Linear Weekly Temperature Range | 0.546 | 0.627 |
| Linear Days(24-30) | 0.551 | 0.628 |
| Linear Days(>30) | 0.549 | 0.627 |
| Linear Days(>24) | 0.557 | 0.63 |
| Linear Days(2-6) | 0.546 | 0.626 |
| Linear Days(<0) | 0.548 | 0.627 |
| Linear Days(<6) | 0.548 | 0.628 |
| Linear Days(<-5) | 0.547 | 0.626 |
| Piecewise Relative Humidity (RH) | 0.557 | 0.633 |
| Piecewise Wind Speed (WS) | 0.557 | 0.633 |
| Piecewise Average Temperature (TAVG) | 0.56 | 0.634 |
| Piecewise TAVG + Piecewise RH | 0.581 | 0.647 |
| Piecewise TAVG + Piecewise RH + Piecewise WS | 0.591 | 0.655 |
| Piecewise TAVG + Piecewise RH + Piecewise WS + Linear Precipitation | 0.598 | 0.657 |
| Piecewise TAVG + Piecewise RH + Piecewise WS + Linear Precipitation + Linear Weekly Temperature Range | 0.599 | 0.658 |
| Piecewise TAVG + Piecewise RH + Piecewise WS + Linear Precipitation + Linear Weekly Temperature Range + Linear Days(<0) + Linear Days (>30) | 0.608 | 0.665 |

**Table S4.** **Summary of regression model performance.** The table shows the performance of eight models, ranging from simple to complex. The best performing model is highlighted in bold.

| **Model** | **Independent Variabels** | **MAE** | **RMSE** | **MSE** | **R2** | **Deviance Explained(%)** |
| --- | --- | --- | --- | --- | --- | --- |
| Linear Regression | Meteorological Factors | 40.99 | 67.93 | 4614.89 | 0.14 | NA |
|  | Air Pollutants | 40.58 | 67.92 | 4613.02 | 0.13 | NA |
| Generalized Linear Regression | Meteorological Factors | 40.06 | 67.27 | 4525.2 | NA | 0.23 |
|  | Air Pollutants | 40.06 | 67.75 | 4589.76 | NA | 0.2 |
| Generalized Additive Model | Meteorological Factors | 35.91 | 63.02 | 3971.24 | 0.26 | 0.37 |
|  | Air Pollutants | 31.76 | 55.91 | 3125.8 | 0.41 | 0.5 |
| Generalized Weighted Quantile Sum Regression | Meteorological Factors | 49.07 | 78.17 | 6110.74 | NA | 0.19 |
|  | Air Pollutants | 49.05 | 78.19 | 6114.07 | NA | 0.25 |
| Distributed Lag Non-Linear Model | Meteorological Factors | 36.02 | 62.44 | 3898.36 | NA | 0.38 |
|  | Air Pollutants | 32.62 | 56.89 | 3236.03 | NA | 0.47 |
| Distributed Lag Non-Linear Model with Gaussian Process | Meteorological Factors | 29.9 | 52.69 | 2776.06 | 0.48 | 0.57 |
|  | Air Pollutants | 28.36 | 50.77 | 2577.48 | 0.51 | 0.61 |
| Distributed Lag Non-Linear Model with Markov Random Field | Meteorological Factors | 26.52 | 46.43 | 2155.93 | 0.6 | 0.65 |
|  | Air Pollutants | 26.3 | 47.64 | 2269.67 | 0.57 | 0.65 |
| **Hierachical Spatial Distributed Lag Non-Linear Model** | **Meteorological Factors** | **26.28** | **45.91** | **2107.84** | **0.61** | **0.66** |
|  | **Air Pollutants** | **26.11** | **47.19** | **2227** | **0.58** | **0.66** |

**Table S5.** **Table of stepwise model selection results for air pollutants**

| **Stepwise Selection** | **R2** | **Explained Deviance (%)** |
| --- | --- | --- |
| Piecewise PM2.5 (WHO) | 0.568 | 0.646 |
| Piecewise SO2 (WHO) | 0.561 | 0.643 |
| Piecewise NO2 (WHO) | 0.558 | 0.638 |
| Piecesie CO (WHO) | 0.570 | 0.650 |
| Piecewise PM2.5 | 0.570 | 0.648 |
| Piecewise NO2 | 0.559 | 0.639 |
| Piecewise CO (WHO) + PM2.5 | 0.579 | 0.656 |
| Piecewise CO (WHO) + PM2.5 + NO2 | 0.579 | 0.659 |
| Piecewise CO (WHO) + PM2.5 + NO2 + SO2 (WHO) | 0.579 | 0.661 |

**Figure S1. Directed acyclic graph of relationships among meteorological factors and RSV infections.** The spatiotemporal factors are highlighted in grey circles, covariates are highlighted in blue circles, and exposures are highlighted in yellow circles. All primary effects that will be investigated are marked by yellow arrows.

Figure S2. Directed acyclic graph of relationships among air pollutants and RSV infections.

**Figure S2. Directed acyclic graph of relationships among air pollutants and RSV infections.** The spatiotemporal factors are highlighted in grey circles, covariates are highlighted in blue circles, and exposures are highlighted in yellow circles. All primary effects that will be investigated are marked by yellow arrows.

**Figure S3.** **Spearman’s rank correlation matrix of weather conditions and respiratory syncytial virus activity in Japan**

**Figure S4. Maps of the geographic distribution of total RSV cases in Japan from 2013 to 2019**

# **Figure S5. Decomposition of weekly RSV cases in Japan from 2013 to 2019 using STL**. The relative size of the grey bars on the right-hand side of each plot shows that the trend is the dominating component, indicating the variation in the seasonal component is small compared to the variation in the trend component.

# **Figure S6. A time-series plot of distinct RSV infection patterns.** (a) Elbow plot for determining the optimal number of clusters (k). (b) A time-series plot of 4 distinct RSV infection patterns. 47 prefectures were clustered into 4 groups by K-Medoids clustering using Dynamic Time Warping (DTW). Here, we used the mean to represent the trend of each cluster, which was calculated by the mean value of the time series within each group.

**Figure S7. Trends in associations between meteorological factors and respiratory syncytial virus activity at different cumulative lags up to 3 weeks.** From (A) to (E), relative humidity, wind speed, total precipitation, average temperature, and temperature range. From (1) to (4), no lag, a cumulative 0-1 week lag, a cumulative 0-2 week lag, and a cumulative 0-3 week lag.


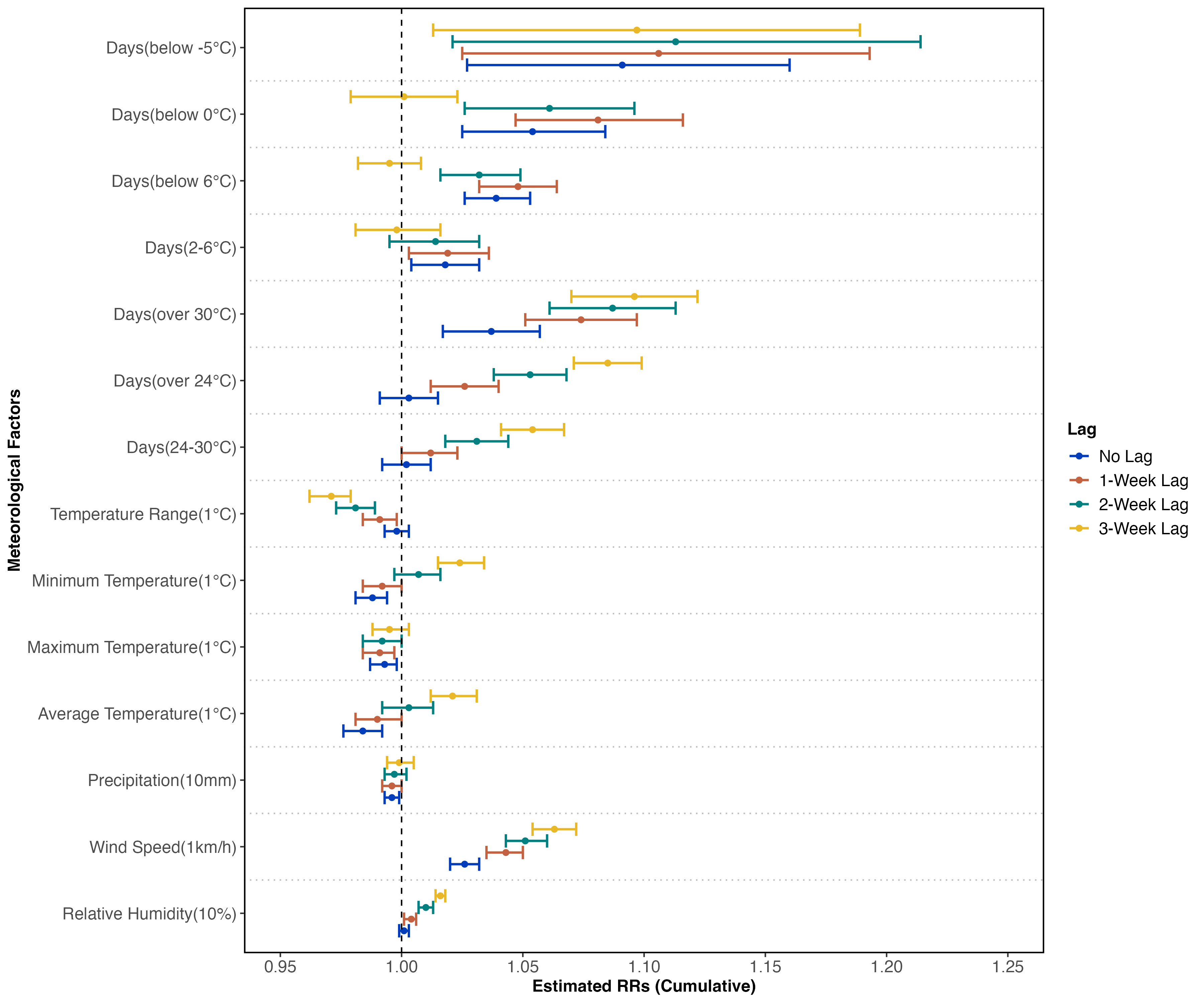


**Figure S8. Plots of linear association between meteorological factors and respiratory syncytial virus activity up to 3 weeks of lag in the adjusted univariate model**

**Figure S9. Plots of association between meteorological factors and respiratory syncytial virus activity up to 3 weeks of lag in the crude univariate model**

**Figure S10. Plots of association between meteorological factors and respiratory syncytial virus activity up to 3 weeks of lag in the adjusted univariate model**

**Figure S11. Plots of subgroup analysis results of average temperature models**

**Figure S12. Trends in associations between air pollutants and respiratory syncytial virus activity at different cumulative lags up to 3 weeks.** From (A) to (D), PM2.5, NO2, CO and SO2. From (1) to (4), a cumulative 0-1 week lag, a cumulative 0-2 week lag, and a cumulative 0-3 week lag.

**Figure S13. Association between air pollutants and respiratory syncytial virus activity up to 3 weeks of lag based on WHO air quality guidelines (2005)**

**Figure S14. Plots of association between air pollutants and respiratory syncytial virus activity up to 3 weeks of lag in the crude univariate model**

**Figure S15. Plots of association between air pollutants and respiratory syncytial virus activity up to 3 weeks of lag in the adjusted univariate model**

**Figure S16. Plots of model sensitivity for changing covariates in meteorological models, up to 3 weeks of lag.** (A) Sensitivity results of no lag. (B) Sensitivity results of a cumulative 1 week of lag. (C) Sensitivity results of cumulative 2 weeks of lag. (D) Sensitivity results of cumulative 3 weeks of lag.

**Figure S17. Plots of model sensitivity for changing lag time in meteorological models.** (A) Sensitivity results of no lag. (B) Sensitivity results of a cumulative 1 week of lag. (C) Sensitivity results of cumulative 2 weeks of lag. (D) Sensitivity results of cumulative 3 weeks of lag.

**Figure S18. Plots of model sensitivity for changing covariates in air pollutant models, up to 3 weeks of lag.** (A) Sensitivity results of no lag. (B) Sensitivity results of a cumulative 1 week of lag. (C) Sensitivity results of cumulative 2 weeks of lag. (D) Sensitivity results of cumulative 3 weeks of lag.

**Figure S19. Plots of model sensitivity for changing lag time in air pollutant models.** (A) Sensitivity results of no lag. (B) Sensitivity results of a cumulative 1 week of lag. (C) Sensitivity results of cumulative 2 weeks of lag. (D) Sensitivity results of cumulative 3 weeks of lag.

**Reference**

1. Air quality guidelines global update 2006. Accessed July 27, 2024. https://www.who.int/publications/i/item/WHO-SDE-PHE-OEH-06.02

2. Victoria EPA. PM2.5 particles in the air | Environment Protection Authority Victoria. Accessed July 27, 2024. https://www.epa.vic.gov.au/for-community/environmental-information/air-quality/pm25-particles-in-the-air

3. Nitrogen dioxide. Ministry for the Environment. Published September 29, 2021. Accessed July 27, 2024. https://environment.govt.nz/facts-and-science/air/air-pollutants/nitrogen-dioxide-effects-health/
